# An autonomous mobile robot path planning strategy using an enhanced slime mold algorithm

**DOI:** 10.3389/fnbot.2023.1270860

**Published:** 2023-10-17

**Authors:** Ling Zheng, Chengzhi Hong, Huashan Song, Rong Chen

**Affiliations:** ^1^School of Electronic Information and Communications, Huazhong University of Science and Technology, Wuhan, China; ^2^Shenzhen Research Institute of Central China Normal University, Shenzhen, China; ^3^State Key Laboratory of Information Engineering in Surveying, Mapping, and Remote Sensing, Wuhan University, Wuhan, China; ^4^Space-Time Information Department, China Mobile Intelligent Mobility Network Technology Co., Ltd., Wuhan, China; ^5^Institute of Traffic Engineering, Wuhan Technical College of Communications, Wuhan, China

**Keywords:** autonomous mobile robots, path planning, slime mold algorithm, dynamic environment, artificial potential field

## Abstract

**Introduction:**

Autonomous mobile robot encompasses modules such as perception, path planning, decision-making, and control. Among these modules, path planning serves as a prerequisite for mobile robots to accomplish tasks. Enhancing path planning capability of mobile robots can effectively save costs, reduce energy consumption, and improve work efficiency. The primary slime mold algorithm (SMA) exhibits characteristics such as a reduced number of parameters, strong robustness, and a relatively high level of exploratory ability. SMA performs well in path planning for mobile robots. However, it is prone to local optimization and lacks dynamic obstacle avoidance, making it less effective in real-world settings.

**Methods:**

This paper presents an enhanced SMA (ESMA) path-planning algorithm for mobile robots. The ESMA algorithm incorporates adaptive techniques to enhance global search capabilities and integrates an artificial potential field to improve dynamic obstacle avoidance.

**Results and discussion:**

Compared to the SMA algorithm, the SMA-AGDE algorithm, which combines the Adaptive Guided Differential Evolution algorithm, and the Lévy Flight-Rotation SMA (LRSMA) algorithm, resulted in an average reduction in the minimum path length of (3.92%, 8.93%, 2.73%), along with corresponding reductions in path minimum values and processing times. Experiments show ESMA can find shortest collision-free paths for mobile robots in both static and dynamic environments.

## 1. Introduction

Autonomous vehicles and mobile robots represent pivotal advancements in modern artificial intelligence, enabling applications across many industries, including manufacturing, agriculture, and healthcare. Developing fully autonomous mobile robot systems requires integrating diverse functionalities, from environmental perception and planning to decision-making, behavior control, and execution. Path planning serves as a foundation for autonomous mobile robot decision-making and controls, especially in intricate and unknown dynamic environments. Optimal path planning algorithms lay the groundwork for safe navigation. However, ensuring safety as robots traverse the real world demands balancing global optimization with local reaction (Wang et al., 2018). Significant research has focused on mobile robot path planning, developing numerous theories and methods addressing its multifaceted challenges (Hewawasam et al., 2022). Path planning presents difficulties due to its non-linear, multi-constraint nature within uncertain environments, making optimal solutions elusive using traditional techniques (Sanchez-Ibanez et al., 2021). Conventional algorithms like the A^*^ (Jin et al., 2022), probabilistic roadmaps (Kavraki et al., 1996), fuzzy reasoning (Liu Z. X. et al., 2022), artificial potential field (Das et al., 2022), and rapidly-exploring random trees (Zhang et al., 2021) have limitations addressing complex environments. Swarm intelligence algorithms excel through self-organizing population interactions informed by mathematical rulesets (Xu et al., 2020). They generate optimal solutions via biological cluster emulation (Rafai et al., 2022). For example, the monarch butterfly optimization (MBO) algorithm simulates butterfly swarms' behavior (Bao et al., 2020). Artificial bee colony (ABC) modeling imitates bee colonies (Liang and Lee, 2015), while the moth flame optimization (MFO) algorithm recreates moths' spiral motions (Zhang et al., 2020). Such bio-inspired techniques offer novel approaches for complex path planning problems (Versaci et al., 2020).

Several studies confirm swarm intelligence's benefits for autonomous mobile robot decentralization (Fragapane et al., 2021). Swarm intelligence algorithms establish mathematical models using rule sets and elements. These algorithms iterate by replacing the current solution with a newly generated one, repeating the optimization process until optimal solutions are obtained or a maximum number of iterations is reached. In a study Cheng et al. (2020), a genetic algorithm (GA) accomplished path planning for a reconfigurable hinged-Tetromino robot, addressing multi-objective global optimization. However, the GA optimization is susceptible to local optima and exhibits slow convergence. To overcome this, an enhanced ant colony optimization (ACO) algorithm incorporating a time taboo grid strategy demonstrated success in dynamic environments (Xiong et al., 2021). However, ACO has a lengthy calculation cycle. Another study applied particle swarm optimization (PSO) with two objective functions—distance and risk—to determine the safest and shortest path by predicting random obstacle changes (Al Hilli et al., 2021). However, PSO is prone to premature convergence in complex problems. An improved cuckoo optimization algorithm (COA) achieved constrained path planning in simulations and real environments through an enhanced objective function (Mohanty, 2020). However, COA left room for improved global convergence efficiency. In a related study Dereli (2022), the whale optimization algorithm (WOA) enhanced convergence by strictly following the leader to prey via Euclidean distance. However, WOA faces frequent local optima challenges. While swarm intelligence algorithms have proven effective in simulations and experiments, opportunities remain to address premature convergence, dynamically balance exploitation/exploration, and overcome localization. Focusing on such areas could further optimize performance. Therefore, improved meta-heuristic path planning for autonomous mobile robots remains an active research direction.

The performance of basic swarm intelligence algorithms generally improved additional components or combining algorithms (Mac et al., 2016). For instance, an enhanced diversity PSO algorithm in the diversity PSO algorithm to ensure diverse peaks and prevent iteration stagnation (Fernandes et al., 2022). Another study combined the bat optimization algorithm with CAO to select optimal qualities, reducing path calculation time (Saraswathi et al., 2018). A new WOA variant incorporated an artificial potential field to improve dynamic obstacle avoidance (Dai et al., 2023). Another approach integrated the Bacterial Foraging Optimization (BFO) algorithm with the Lévy flight to reduce iterations and accelerate convergence (Pang et al., 2019). Additionally, the gray wolf optimization algorithm (GWO) combined with PSO in the PSO_GWO introduced PSO to calculate gray wolf positions, effectively addressing local optimization (Teng et al., 2019). Moreover, meta-heuristic optimization processes are inherently stochastic—striking the right balance between exploration and exploitation proves crucial in optimizing algorithms (Mirjalili et al., 2014).

The slime mold algorithm (SMA) is a meta-heuristic inspired by slime mold behavior in nature (Li et al., 2020). It exhibits fewer parameters, robustness, and exploration ability, making it promising for global optimization. In benchmark function sets like IEEE CEC 2014, SMA outperformed algorithms such as the WOA, MFO, and GWO. However, SMA also demonstrates weaknesses, such as slow convergence or inability to reach global optima in certain states or functions. Like all algorithms, SMA has limitations in specific scenarios or problem types. Regarding path planning, SMA surpasses particle swarm optimization (PSO) and artificial bee colony (ABC) algorithms in static environments (Agarwal and Bharti, 2021). Further research is needed to address dynamic environment challenges. Improving convergence efficiency and achieving effective dynamic obstacle avoidance are critical for mobile robot path planning. Dedicated efforts developing innovative techniques and algorithms that can adapt swiftly to dynamic changes are needed.

To improve the efficiency and effectiveness of autonomous mobile robot path planning in dynamic environments and achieve the best paths, this paper introduces an enhanced version of the Slime Mold Algorithm (ESMA). The proposed planning process involves three stages: (1) Processing the navigation environment to create a movement position map for the robot, (2) Planning the trajectory between the start and endpoint of the desired movement, and (3) Post-processing the determined path to ensure the shortest route while considering the inherent limitations of the autonomous mobile robot. The final stage will not be discussed in this article. The main contributions of this study can be summarized as follows:

a) Adaptive technology using a linear decreasing strategy to increase random search and enhance the global capability.
b) Improved potential field factor incorporating attraction and repulsion for dynamic obstacle avoidance.

The structure of this paper is as follows: Section 2 provides an overview of the enhanced Slime Mold Algorithm (ESMA). Section 3 presents the underlying theory of the SMA and its improvement. Section 4 conducts experiments demonstrating ESMA's dynamic path planning benefits for mobile robots. Finally, Section 5 summarizes key conclusions regarding the significance of the findings.

## 2. Related work

Like other meta-heuristic algorithms, the SMA involves three key aspects when dealing with optimization: exploration, exploitation, and the transaction between the two stages (Cai et al., 2020). Exploration dedicates to identifying potential areas within the entire search space that may contain the optimal solution, aiming to progressively narrow this region. Conversely, exploitation focuses on finding the best solution within the defined feasible area (Alyasseri et al., 2022). The smooth transition between the exploration and exploitation stages plays a pivotal role in achieving a well-balanced search strategy for the algorithm (Rodriguez-Molina et al., 2020). Optimizing exploration and exploitation strategies provide an important approach for swarm intelligence algorithm optimization (Lin and Gen, 2009).

A series of proposed improved SMAs have addressed the issues of optimization and slow convergence while also considering obstacle avoidance in the path planning. Yu et al. (2021) proposed a strategy combining exploration and exploitation using quantum rotation gates and a water cycle approach to enhance convergence speed. To mitigate premature convergence (Rizk-Allah et al., 2022), implemented a strategy incorporating chaotic search and cross-reverse enhancement, effectively expanding the search space and improving non-linear convergence accuracy. Researchers introduced reverse learning to improve global exploration ability. Liu and Liu (2022) combined quasi-reverse learning and quasi-reflective learning techniques to enlarge population searching range. Additionally, they utilized the unscented transformation sigma point to improve stability and alleviate stagnation. Nguyen et al. (2020) proposed an enhanced SMA incorporating opposition-based learning and adjusting individual position update weight coefficients to enhance performance. Houssein et al. (2022) integrated modified opposition-based and orthogonal learning techniques to improve accuracy. These studies demonstrated advantages in balancing exploration and exploitation stages. However, configuring the reverse learning search space poses a challenge directly impacting effectiveness.

The crossover and mutation operators in the adaptive guided differential evolution algorithm (AGDE) possess strong local optimization capabilities, making them effective for enhancing the local search capability of SMA. Houssein et al. (2021) utilized AGDE to promote population diversity and overcome premature convergence, resulting in SMA-AGDE included here for comparison. The Lévy flight strategy is a well-known approach for improving global search ability in intelligent optimization algorithms (Liu J. X. et al., 2022). Building on the Lévy search (Zheng et al., 2023), introduced rotation perturbation with local optimization ability, yielding the Lévy flight-rotation SMA (LRSMA) with higher convergence accuracy. LRSMA proves particularly effective for static environment path planning problems. Consequently, LRSMA is also included as one of the comparative algorithms in this study.

While these improved SMAs can enhance the algorithm's convergence speed, further research on obstacle avoidance in dynamic environments is needed. Hence, this study proposes an ESMA considering the environment in two dimensions and efficiently plan autonomous mobile robot paths dynamically. To verify the efficiency and utility of the ESMA, experimental comparative studies evaluated it against SMA, SMA-AGDE, and LRSMA.

## 3. Mathematical model of algorithm

### 3.1. SMA and its advanced approach

#### 3.1.1. SMA

The SMA abstracts and simulates the foraging behavior and morphological changes observed in slime molds through a mathematical model. During foraging, the SMA creates a venous network that connects various food sources. The higher the quality and density of the food source, the thicker the venous network. As slime molds locate food, the SMA dynamically adjusts the cytoplasm flow rate within veins and modifies network thickness using oscillations from a biological oscillator. When food concentration is higher, the wave generated by the biological oscillator becomes stronger, resulting in faster cytoplasm flow and thicker veins.

The SMA maintains a balance between exploration and exploitation throughout the search process. Even after finding food sources, there remains a probability for slime molds to explore unknown areas, ensuring the algorithm continues searching for potentially better solutions. The search mechanism of the SMA can be summarized as follows (Li et al., 2020):


(1) $$\begin{array}{l} X\left(t+1\right)\\ \;=\left\{ \begin{array}{l} rand\times \left(UB-LB\right)+LB,\;rand<z\\ \;X\_best\left(t\right)+vb\\ \times \left(W\times X_{r1}\left(t\right)-X_{r2}\left(t\right)\right),\;rand\geq z\;and\;rand_{1}<p\\ \;vc\times X\left(t\right),\;rand\geq z\;and\;rand_{1}\geq p \end{array}\right. \end{array}$$

(2) $$\begin{array}{l} W\left(SIndex(i)\right)\\ \;=\left\{ \begin{array}{l} 1+r_{2}\times \mathrm{lg}\left(\frac{bF-S(i)}{bF-wF}+1\right),\;condition\\ 1-r_{2}\times \mathrm{lg}\left(\frac{bF-S(i)}{bF-wF}+1\right),\;others \end{array}\right. \end{array}$$

(3) $$\begin{array}{l} SIndex\left(i\right)=sort\left(N\right) \end{array}$$

In the SMA, the following equations and parameters are utilized:

*t*: Current iterative index.

*X*_*r*1_(*t*) and *X*_*r*2_(*t*): Positions of two randomly selected individuals from the slime molds.

*X*(*t*+1): Updated position of the individual after the current iteration.

*X*_*best*(*t*): Position of the individual with the highest food concentration in the tth iteration (optimal position).

*vc*: Control parameter measuring the utilization of historical records by individual slime molds, decreasing linearly in the range of [1, 0].

*UB* and *LB*: Upper and lower bounds of the search space, respectively.

*rand*: Random number between 0 and 1.

*rand*_1_: Random number between 0 and 1, representing the switching probability between exploration and exploitation modes.

z: The proportion of slime molds randomly distributed in all populations.

*W*: Weight coefficient, simulating the change in the biological oscillator's frequency with the quality and density of the food during foraging.

*r*_2_: Random number, either 0 or 1.

*SIndex*(*i*): Index of slime mold individuals after sorting.

*bF* and *wF*: Optimal and worst fitness values in the current iteration, respectively.

lg: Value used to slow down the rate of change.

*condition*: Slime mold individuals whose fitness values are in the top half of the population.

*others*: Remaining slime mold individuals.

The exploration stage is active when *rand*_1_<*p*, while the exploitation stage occurs when *rand*_1_≥*p*.

*P* and *vb* are control parameters, and the formula is as follows:


(4) $$\begin{array}{l} p=\tanh|S\left(i\right)-DF|,\;i\in \left\{1,2,\ldots ,N\right\} \end{array}$$

(5) $$\begin{array}{l} a=arctanh\left(-\frac{t}{IT_{\max}}+1\right) \end{array}$$

*i*: Index of slime molds.

*S*(*i*): Fitness value of the *i*th slime mold in the current iteration.

*DF*: Optimal fitness value among all iterations.

*vb*: Random value in [–a, a], with the range decreasing as the value of *a* decreases.

*IT*_max_: Maximum number of iterations.

**Figure 1:**
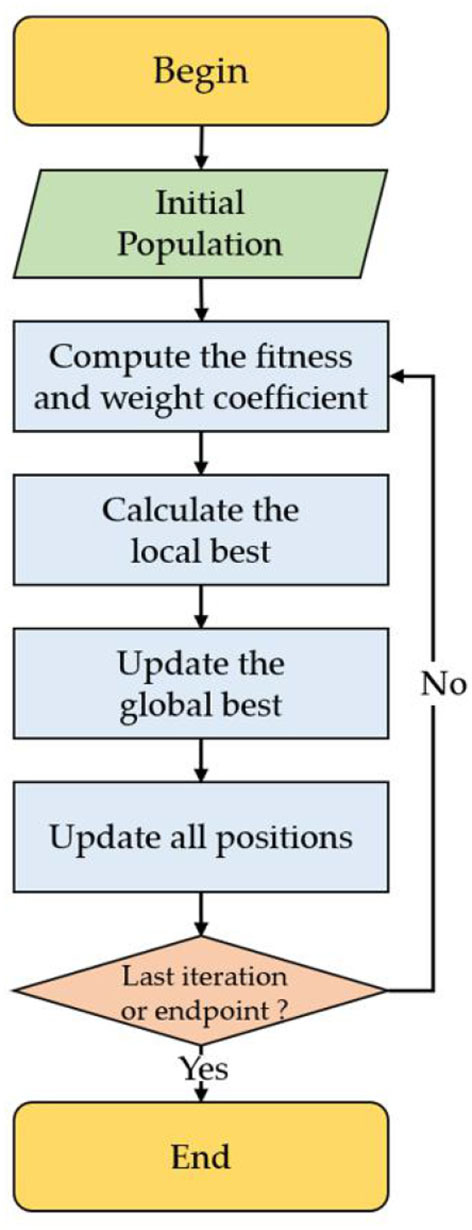
The diagram of the slime mold algorithm (SMA).

Figure 1 shows the SMA detailed steps, which involves the following processes:

(1) Initialization.
(2) Obtain each individual's position through random search, calculating corresponding fitness values, and local best solution.
(3) Renew the global best consequence.
(4) Determine whether the maximum search iteration limit is reached. If so, continue updating individuals based on value and Equation (1) until the best solution is obtained.

#### 3.1.2. SMA-AGDE

AGDE demonstrates robust convergence ability through mutation, crossover and selection (Mohamed and Mohamed, 2019). In the SMA-AGDE, the initial population forms via random sampling, hybridizing SMA and AGDE. AGDE's mutation and crossover process serves as SMA's individual update method. This integration aims to increase diversity, enhance local search capability, and prevent premature convergence. By combining SMA and AGDE strengths, the SMA-AGDE achieves improved performance in exploration, exploitation, and overall optimization.

**Figure 2:**
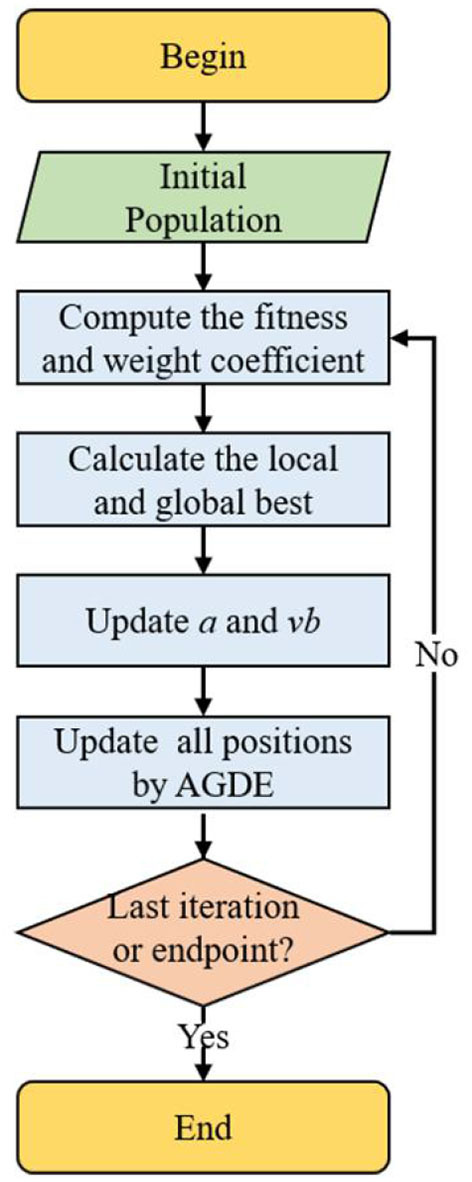
The flow chart of the SMA combined with adaptive guided differential evolution (SMA-AGDE).

Figure 2 illustrates the SMA-AGDE search update mechanism. The main steps are (Houssein et al., 2021):

(1) Initialization.
(2) Obtain each individual's position through random search, calculate fitness value, and find local best solution.
(3) Update parameters *a* and *vb*.
(4) Update the slime mold individuals according to AGDE.
(5) Repeat steps (2)–(4) until the best solution occurs when iterations reach the maximum limit.

#### 3.1.3. LRSMA

The LRSMA updates slime mold positions by introducing a variable neighborhood Lévy flight strategy to improve the global optimization ability of the SMA. The LRSMA applies a rotation disturbance mutation approach considering local optimization tolerance to disturb each slime mold, expand population local search range, and enhance algorithm development ability.

**Figure 3:**
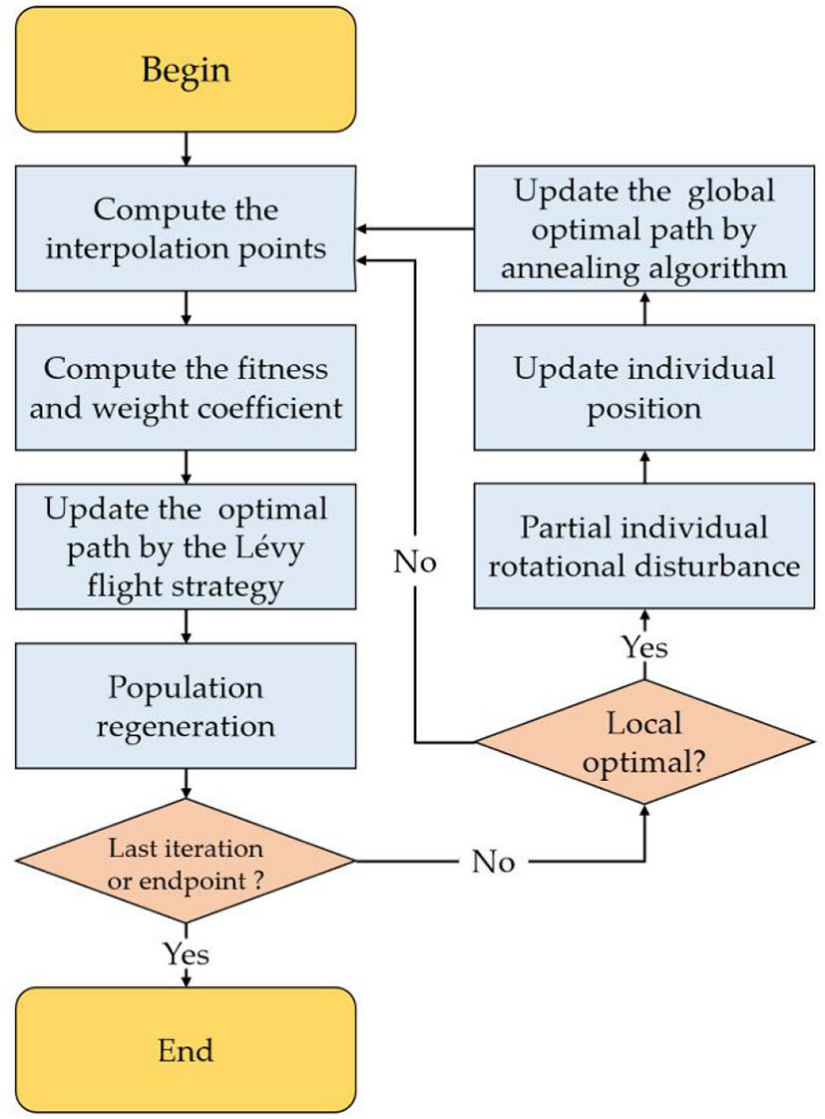
The process description of the LRSMA.

Figure 3 shows the diagram of the LRSMA (Zheng et al., 2023), whose steps are as follows:

(1) Initialize.
(2) Calculate the global best solution. Calculate and sort fitness values to obtain the best and worst fitness value. Update each individual's weight coefficient *W*(*SIndex*) and position.
(3) Re-update global best solution. Update optimal position according to variable neighborhood Lévy flight strategy.
(4) Reconstruct convergence population. Judge the algorithm convergence. If converged, rotate some individuals. Recalculate the global best position by simulating annealing approach.
(5) Regenerate the population.
(6) Repeat steps (2)–(5) until the iteration number reaches the maximum limit and output the global best solution.

### 3.2. The proposed ESMA

The ESMA proposes a new adaptive technique that utilizes the same weight coefficient processing as SMA. The ESMA uses the same fitness calculation as LRSMA in path planning. However, the ESMA's data update method after local optimization differs from LRSMA.

The free space method models the autonomous mobile robot's driving environment, where achievable space is blank and unachievable space contains obstacle area. To simplify calculation, a point-like object moving on a two-dimensional platform simulates the robot. Obstacles enlarge to half robot width on mapping to the environment, represented by circles of radius determining obstacle space (Na et al., 2022).

This paper aims to search the drivable area in the free space through the algorithm to obtain an effective start-end trajectory plan. The trajectory consists of discontinuous points forming a continuous shortest path on the map. This process solves three problems: obstacle avoidance, global path obtaining, and local path planning (Dai et al., 2023).

In the SMA, *z* represents the proportion of randomly distributed slime mold individuals among the total population. As iterations increases, *z* remains constant. Early on, the SMA quickly approaches the best position of the current population. However, when falling into local optimization, it may jump from local optima through random search and explore unknown regions with certain probability. Since *z* directly determines random search probability, the fixed parameter *z* cannot fully meet the search requirements. To address this, an adaptive technique linearly increases *z* within a constrained interval as iterations increase. The adaptive update process of *z* is as follows:


(6) $$\begin{array}{l} z=z_{0}^{*}\left(1+\frac{t}{IT_{\max}}\right) \end{array}$$

where, *z*_0_ is a fixed parameter, *t* is the current iteration, and *IT*_max_ is the maximum number of iterations. The value of *z* is the smallest in the early stage of the search, and the value of *z* becomes larger in the later stage of the search.

In the later stage of the SMA, the population tends to converge to the best individual, which reduces diversity and increasing susceptibility to local optimization. When SMA falls into local optimization, adjusting the parameter z alone may be insufficient for escape. In the ESMA, gravitational and repulsive potential fields are established. A synthetic potential field model is constructed based on these, on these, combining the strengths. The gravitational field improves local planning efficiency while the repulsive field enables obstacle avoidance in local robot path planning. The introduced potential integrated field serves as another ESMA optimization variable.

**Figure 4:**
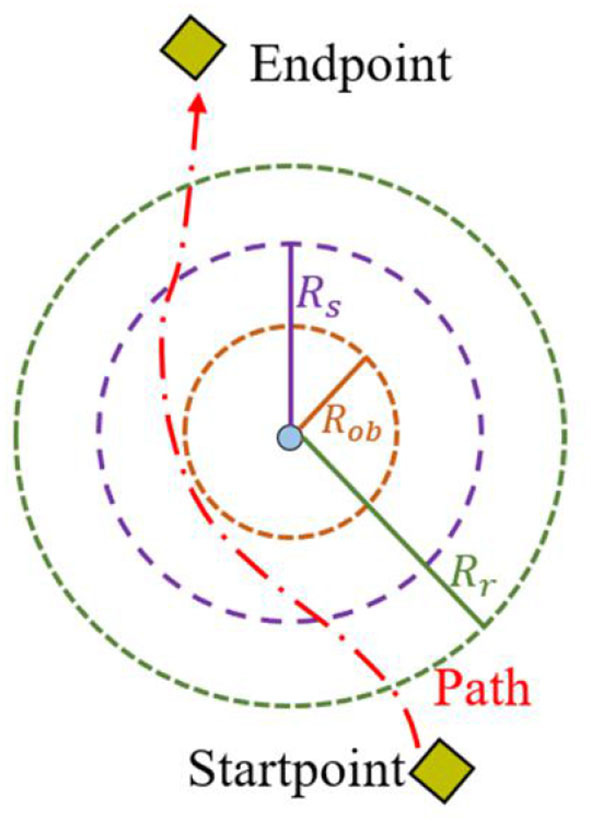
The schematic diagram of the annular synthetic magnetic field. Two yellow diamonds represent the starting point and endpoint. Red dashed line stands for the path of the robot. *R*_*s*_ is the range of the gravitational attenuation zone; *R*_*ob*_ is the threat range of the obstacle; *R*_*r*_ is the range of the repulsion transition zone.


Traditional artificial potential fields suffer from issues such as unreachable targets and local minimum traps. Therefore, this paper employs a circular synthesis-enhanced potential field method to construct a comprehensive potential field model that neglects long-range repulsion and weakens the attraction from obstacle points. As shown in Figure 4, the potential synthetic field in the annular region can provide a non-oscillatory trajectory (Liu et al., 2020). In the robot local path planning process, the repulsive potential field is weakened when the robot is far from obstacles to prevent premature deviation from the trajectory. Simultaneously, the attractive potential field is weakened when approaching obstacles to prevent local oscillations. The synthetic potential in the annular region is given by Liu et al. (2020).

(7) $$\begin{array}{l} U\left(X\right)=\varepsilon_{a}{*U}_{a}\left(X\right)+\varepsilon_{r}*U_{r}\left(X\right) \end{array}$$

(8) $$\left\{ \begin{array}{l} \;U_{a}\left(X\right)=-\eta_{a}*{\left(1-\frac{d\left(X,X_{g}\right)}{d\left(X_{ob},X_{g}\right)}\right)}^{q_{a}}\\ \eta_{a}=\left\{ \begin{array}{l} \;0,\;d\left(X,X_{ob}\right)<R_{ob}\\ \frac{1}{2}\left(\sin\left(\frac{d\left(X,X_{ob}\right)-R_{ob}}{R_{s}-R_{ob}}\cdot \pi -\frac{\pi }{2}\right)+1\right),\;R_{ob}\leq d\left(X,X_{ob}\right)<R_{s}\\ \;1,\;otherwise \end{array}\right. \end{array}\right.$$

(9) $$\left\{ \begin{array}{l} \;U_{r}\left(X\right)=-\eta_{r}*{\left(\frac{R_{ob}}{d\left(X,X_{ob}\right)}\right)}^{q_{r}}\\ \eta_{r}=\left\{ \begin{array}{l} \;1,\;d\left(X,X_{ob}\right)<R_{s}\\ \frac{1}{2}\left(\cos\left(\frac{d\left(X,X_{ob}\right)-R_{s}}{R_{r}-R_{s}}\cdot \pi \right)+1\right),\;R_{s}\leq d\left(X,X_{ob}\right)<R_{r}\\ \;0,\;d\left(X,X_{ob}\right)\geq R_{r} \end{array}\right. \end{array}\right.$$

where *U*(*X*) is the potential synthetic field of the robot at position *x*; ε_*a*_ is the gravitational potential field coefficient; *U*_*a*_(*X*) is the potential gravitational field; *U*_*r*_(*X*) is the potential repulsive field; ε_*r*_ is the coefficient of the repulsive potential field; η_*a*_ is the gravitational field coefficient; *X*_*ob*_ is the obstacle position; *X*_*g*_ is the end position; *q*_*a*_ is a positive integer; *R*_*ob*_ is the threat range of the obstacle; *R*_*s*_ is the range of the gravitational attenuation zone; *d*( ) is the Euclidean distance of two points; η_*r*_ is the repulsive field coefficient; *q*_*r*_ is a positive integer; *R*_*r*_ is the range of the repulsion transition zone.

To smoothly navigate around obstacles and avoid local oscillations, improvements have been made to the attractive potential field function by introducing a weakened attractive potential field region near obstacles. To prevent premature deviation from the desired path, the repulsive potential field has been enhanced through a potential field smoothing transition strategy. The potential gravitational function *F*_*a*_(*X*) and the potential repulsive function *F*_*r*_(*X*) can be obtained by deriving the gravitational and repulsive potential functions, respectively. The mathematical models of gravity and repulsion are as follows:


(10) $$\begin{array}{l} \;F_{a}\left(X\right)\\ =\left\{ \begin{array}{l} 0,\;d\left(X,X_{ob}\right)<R_{ob},\\ \;\frac{1}{2}\left(\sin\left(\frac{d\left(X,X_{ob}\right)-R_{ob}}{R_{s}-R_{ob}}\cdot \pi -\frac{\pi }{2}\right)+1\right)*\frac{q_{a}}{d\left(X_{ob},X_{g}\right)}*\\ \;{\left(1-\frac{d\left(X,X_{g}\right)\left\|X-X_{g}\right\|}{d\left(X_{ob},X_{g}\right)}\right)}^{q_{a}-1},\;R_{ob}\leq d\left(X,X_{ob}\right)<R_{s},\\ \frac{q_{a}}{d\left(X_{ob},X_{g}\right)}*{\left(1-\frac{d\left(X,X_{g}\right)}{d\left(X_{ob},X_{g}\right)}\right)}^{q_{a}-1},\;otherwise, \end{array}\right. \end{array}$$

(11) $$F_{r}\left(X\right)=\left\{ \begin{array}{l} \;\frac{q_{r}*R_{ob}{}^{q_{r}}}{d^{q_{r}+1}\left(X,X_{ob}\right)},\;d\left(X,X_{ob}\right)<R_{s},\\ \frac{1}{2}\left(\cos\left(\frac{d\left(X,X_{ob}\right)-R_{s}}{R_{r}-R_{s}}\cdot \pi \right)+1\right)*\frac{q_{r}*R_{ob}{}^{q_{r}}}{d^{q_{r}+1}\left(X,X_{ob}\right)},\;R_{s}\leq d\left(X,X_{ob}\right)<R_{r},\\ \;0,\;d\left(X,X_{ob}\right)\geq R_{r}, \end{array}\right.$$

where *F*_*a*_(*X*) represents gravity, and *F*_*r*_(*X*) represents repulsion.

The directions of gravity and repulsion correspond to the fastest decreasing directions of potential gravitational and repulsive energies, respectively, represented by the negative gradients of the potential gravitational field and potential repulsion field. The resultant force acting on the robot can be obtained via vector superposition of the gravity and repulsion acting on it.

In this paper, the robot's position changes detect local optimization presence in the mobile robot. When the optimal solution of the robot oscillates near a certain point within a small range, it is considered to have fallen into the local optimization. Hence, it is necessary to guide the robot with potential synthetic field local planning until escaping local optimization.

Algorithm 1 shows the ESMA pseudocode.

**Algorithm 1:** The pseudocode of the ESMA.

1: Set the obstacle avoidance threshold
*D*_*min*_ and initialize the population
2: ***for*** *it* =:*IT*_max_ ***do***
3: ***for*** each individual ***do***
4: Calculate the fitness value of each
individual
5: Update the local best solution
6: ***end for***
7: Recalculation the global best solution
8: ***if*** the global best solution reaches the
endpoint ***then***
9: ***return*** the global best solution
10: ***end if***
11: ***if*** *d*(*X*_*best*_(*t*), *X*_*best*_(*t*−1)) < *D*_*min*_ ***then***
12: ***repeat*** add potential field and
update the global best solution
13: ***until*** *d*(*X*_*best*_(*t*), *X*_*best*_(*t*−1))≥*D*_*min*_
14: ***end if***
15: Check if any individual goes out of
search space and modify it
16: Calculate the fitness of each
individual
17: Update the population
18: ***end for***
19: ***return*** the global best solution

The steps of the ESMA are as follows:

(1) Initialization. Randomly generate the initial myxobacteria population and update the fitness value of each smile mold individual.
(2) Update the local and global best solutions according to Equations (1)–(6).
(3) If the algorithm falls into the local optimization, re-update the global best solution according to Equations (7)–(11) and proceed to step (4). Otherwise, proceed to step (4).
(4) Update the population.
(5) Continue iterating through steps (2)–(4) until the best solution is obtained, as long as the number of searches is less than the specified number of iterations.

## 4. Experiment results and analysis

### 4.1. Scenarios and parameter setting

**Figure 5:**
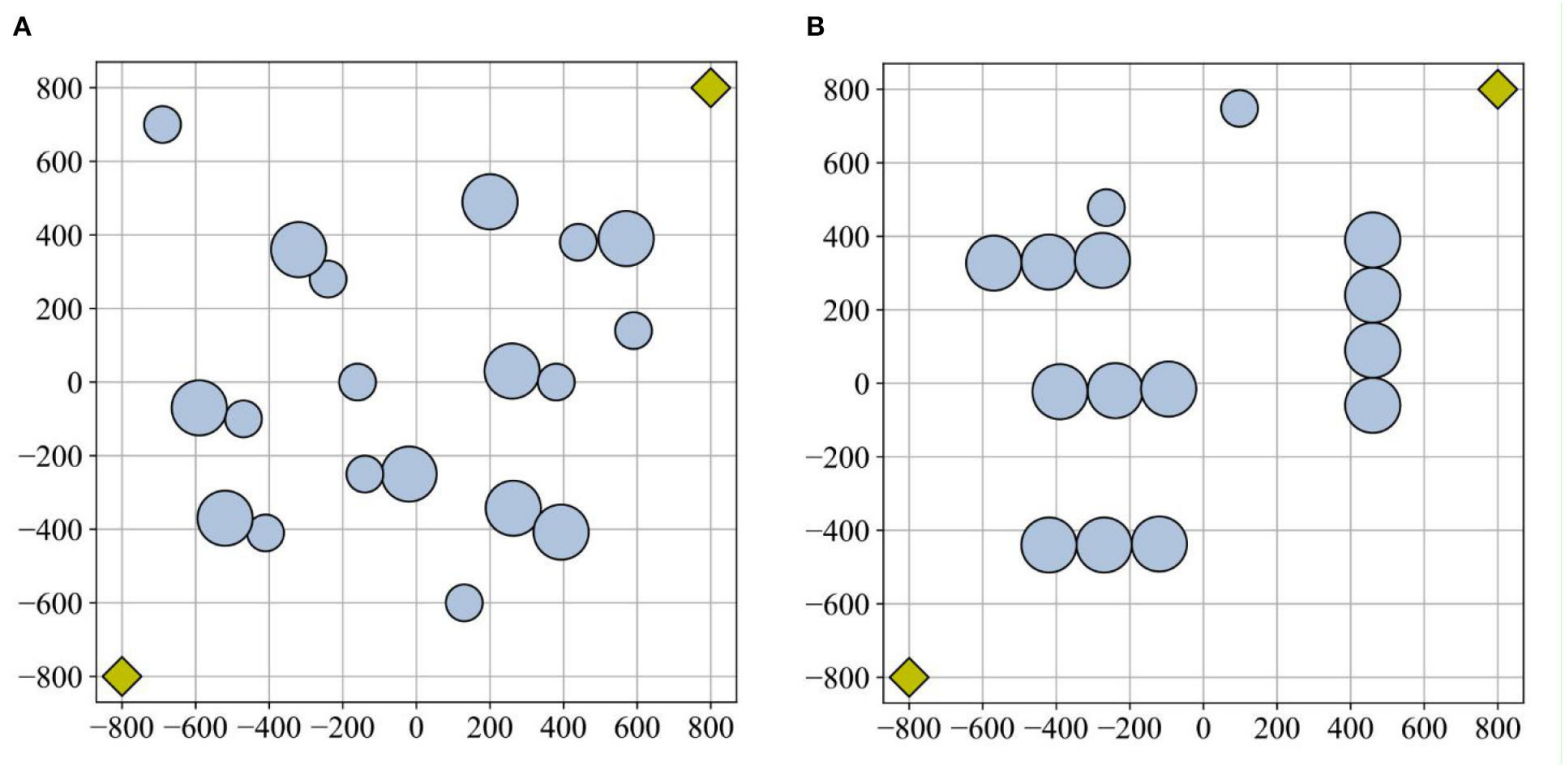
Experimental scenarios: **(A)** scenario 1 and **(B)** scenario 2. The obstacle area is depicted by a blue circle. The starting point and endpoint coordinates are represented by a yellow diamond.

The experimental scenario simplified the obstacles as circles with different radii. To represent the position of the robot, the coordinates of its center point were utilized, irrespective of its size. To verify the algorithm's robustness, two scenarios were designed, as shown in Figure 5, with different obstacle distributions. In Figure 5, the obstacles are punctate in scenario 1, while in scenario 2, they have a banded distribution. The experimental area is 800^*^800 cm, and the white grid represents the accessible area.

The obstacle area is depicted by a blue circle, where the size of the circle corresponds to the extent or coverage of the obstacle. Obstacles with larger radii indicate larger obstacles. The radii are 75 and 50 cm. The starting point and endpoint coordinates are [−800, −800] and [800, 800], respectively, represented by a yellow diamond. The unit of measurement is cm.

To provide a comprehensive evaluation of the algorithm's performance, this study conducted comparisons between the ESMA and other algorithms, namely SMA (Agarwal and Bharti, 2021), SMA-AGDE (Houssein et al., 2021), and LRSMA (Zheng et al., 2023). The parameter settings for all algorithms were determined based on the reference papers. In SMA, the parameter is *z* = 0.3. The parameters of SMA-AGDE are set as *z* = 0.3, *CR*1∈[0, 0.05], *CR*2∈[0.9, 1.0]. In LRSMA, the parameters are *z* = 0.3, *Fmin* = 1, *p*_*r*_ = 0.5. The parameters of ESMA are set as *z*∈[0.3, 0.6], *Fmin* = 1, *D*_*min*_ = 10, *q*_*a*_ = 1, *q*_*r*_ = 1, *R*_*ob*_ = 200, *R*_*s*_ = 250, *R*_*r*_ = 400. The evaluation criteria in this study included path length, planning time, and planning success rate. The minimum value, average value, and standard deviation of these metrics were calculated and compared to assess the algorithm's performance across these indices.

### 4.2. Simulation experiment and analysis

#### 4.2.1. Experimental environment

The running environment of the simulation experiment platform was as follows: the CPU was an Intel Core i7-6500U, the frequency was 2.50 GHz, the memory was 8 GB, the programming language was Python 3.9, the operating system was Windows10 64 bit, and the compilation software was Visual Studio Code 1.6.1.

#### 4.2.2. Simulation experiments and analysis

The experiments conducted in static environments involved the application of the SMA, SMA-AGDE, LRSMA, and ESMA algorithms to two environments as shown in Figure 5. The algorithm's universality was confirmed through 100 simulation experiments. Record the data for 100 successful runs (excluding local optima), including the optimal path length and processing time. Also, calculate the success rate in a 100-run algorithm. The path length serves as a reflection of the algorithm's ability to find the optimal path, with shorter lengths indicating better solutions. Optimization time refers to the time taken by the algorithm to find the optimal path. Analyzing the data from 100 experimental runs allows for the calculation of metrics such as the minimum path length for the best solution, the average path length, the standard deviation of the path length, the time required to process the best solution, and the average processing time.

**Figure 6:**
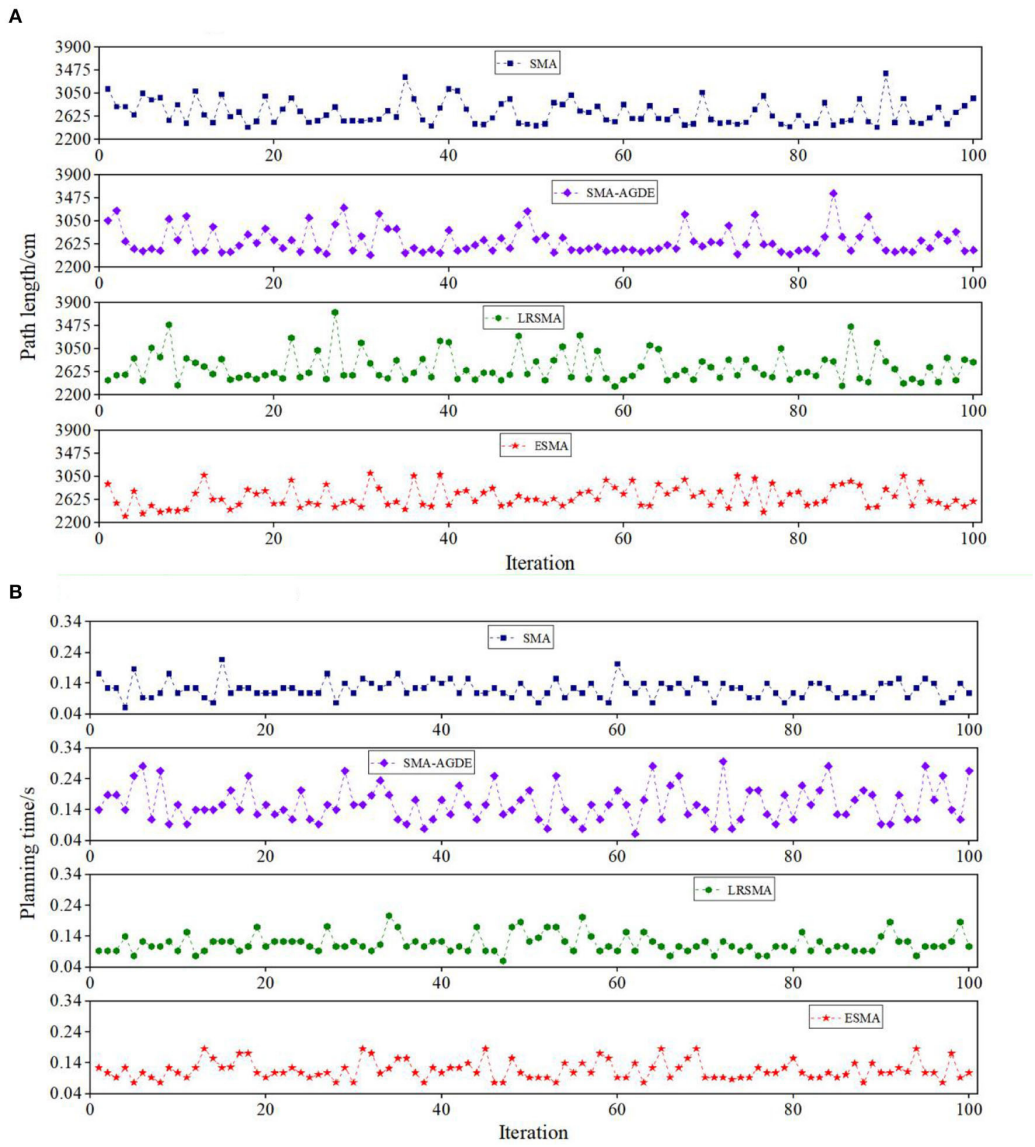
Detail simulation performance of the four algorithms in scenario 1: **(A)** path length and **(B)** planning time. The Y coordinate of the shape is the path length in **(A)** and the planning time in **(B)**. The result is the record of 100 runs in each picture.

Table 1 provides a performance analysis of these four algorithms. Figure 6 shows detailed results of the four algorithms in 100 experiments conducted in scenario 1. In terms of the path length, from Figure 6A, it can be observed that the ESMA algorithm yields the shortest path, measuring 2,323 cm, with the least overall fluctuation. The SMA-AGDE algorithm, on the other hand, results in the longest optimal path, measuring 2,423 cm. LRSMA exhibits the most significant fluctuations in path length. Table 1 reveals that the standard deviation of path lengths for ESMA is 194, while for LRSMA, it is 276. The ESMA outperformed the SMA, SMA-AGDE, and LRSMA. The evaluation indicators, i.e., minimum path length and average path length, of the ESMA decreased by (4.26, 4.30, and 1.59%) and (0.71, 0.22, and 1.38%), respectively. During the search process, the APF method is employed to guide the algorithm in breaking free from local optimization and directing it toward the desired endpoint. In situations where the SMA becomes trapped in local optimization, the ESMA algorithm addresses this issue by increasing the proportion of the random search population and incorporating the APF technique. From Figure 6B, it can be observed that the ESMA algorithm requires the least amount of time, which is 0.093 in terms of time. From Table 1, it can be observed that the ESMA showed a significant improvement over the SMA, SMA-AGDE, and LRSMA, with the two evaluation indexes of the ESMA (the time to process the best solution and the average time) decreasing by (33.33, 67.74, and 16.13%) and (3.42, 36.75, and 0.00%), respectively, indicating that the ESMA has the fastest convergence speed. The results suggest that ESMA is capable of generating faster, more stable, collision-free, and shorter paths.

**Table 1:** Simulation performance of the four algorithms.

		**Path length (cm)**			**Planning time (s)**		**Satisfaction**
		**Minimum**	**Mean**	**Standard deviation**	**Processing the best solution**	**Mean**	**Rate**
Scenario 1	SMA	2,422	2,695	221	0.124	0.121	45%
	SMA-AGDE	2,423	2,682	240	0.156	0.160	50%
	LRSMA	2,360	2,713	276	0.108	**0.117**	65%
	ESMA	**2,323**	**2,676**	**194**	**0.093**	**0.117**	**98%**
Scenario 2	SMA	2,493	2,726	153	**0.077**	0.118	40%
	SMA-AGDE	2,485	2,943	299	0.172	0.180	56%
	LRSMA	2,492	2,723	**139**	**0.077**	0.115	60%
	ESMA	**2,465**	**2,717**	142	**0.077**	**0.114**	**85%**

Best results are shown in bold.

**Figure 7:**
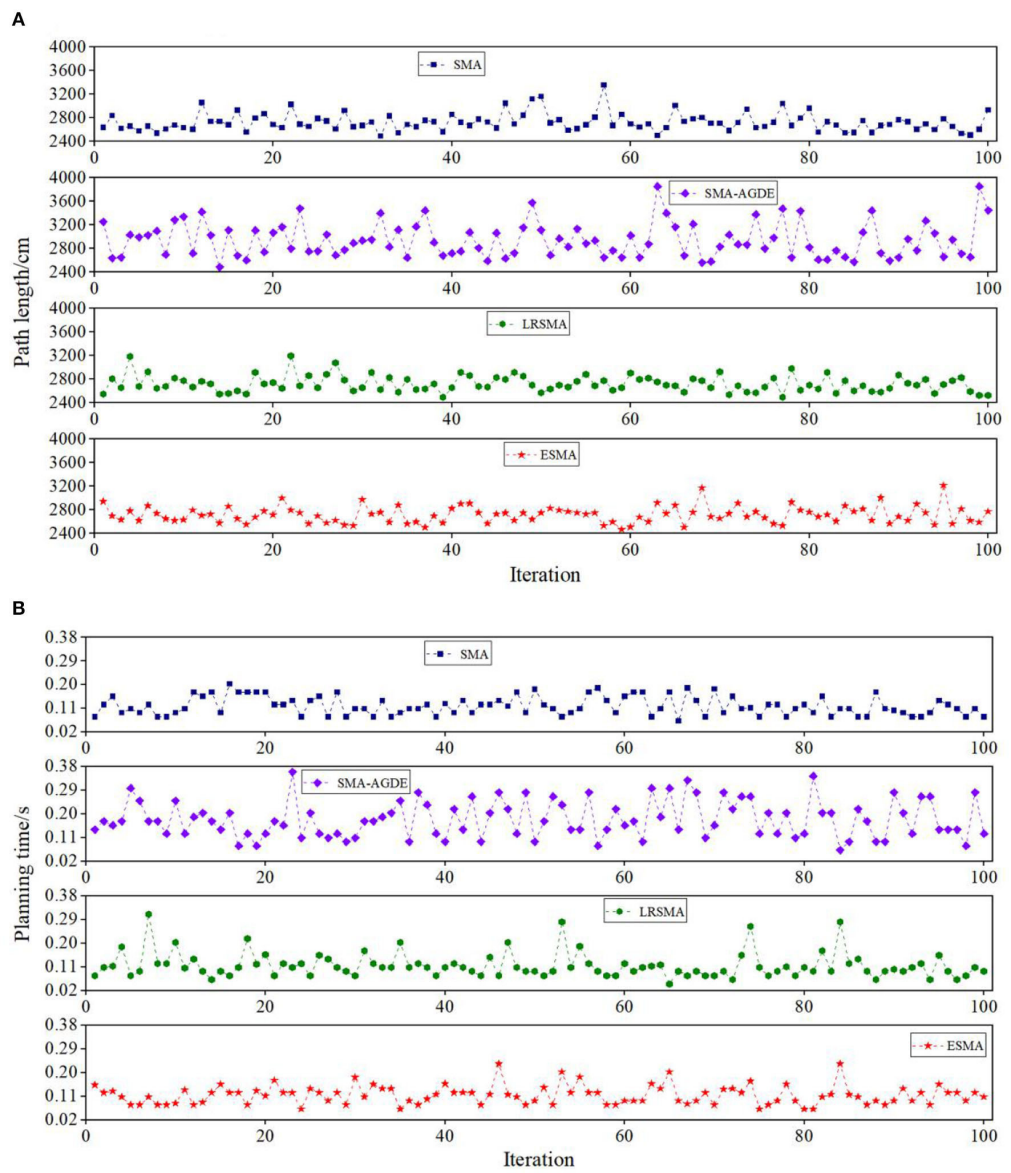
Detail simulation performance of the four algorithms in scenario 2: **(A)** path length and **(B)** planning time. The Y coordinate of the shape is the path length in **(A)** and the planning time in **(B)**. The result is the record of 100 runs in each picture.

Figure 7 provides a detailed breakdown of the results obtained from the 100 experiments conducted in scenario 2, showcasing the performance of the four algorithms. When comparing the path lengths, from Figure 7A, it can be observed that the ESMA algorithm yields the shortest path, measuring 2,465 cm, with relatively minor overall fluctuations compared to LRSMA. The SMA algorithm, on the other hand, results in the longest optimal path, measuring 2,493 cm. SMA-AGDE exhibits the most significant fluctuations in path length. From Table 1, the ESMA algorithm demonstrated significant improvements over the SMA, SMA-AGDE, and LRSMA algorithms. The minimum and average path lengths achieved by ESMA were reduced by approximately (42.7, 18.2, and 2.1%) and (5.3, 2.0, and 1.5%), respectively, compared to the other algorithms. The standard deviation of the path length of the ESMA was larger than that of the LRSMA and smaller than that of the SMA and SMA-AGDE. This phenomenon is due to the fact that the adaptive parameters of the ESMA increase the randomness of the algorithm, while the APF strategy in the ESMA algorithm enhances its global search ability. When comparing the data from Figure 7B, it can be observed that the processing time for obtaining the best solution was comparable between ESMA, SMA, and LRSMA, while SMA-AGDE required a longer processing time. From Table 1, it can be seen that the minimum required time for ESMA, SMA, and LRSMA algorithms is all 0.077, while SMA-AGDE requires a time of 0.172. The average time was slightly better than that of the LRSMA and SMA, and less than that of the SMA-AGDE. However, when the obstacles are distributed in a banded pattern, it becomes necessary to increase the amount of continuous APF guidance, thus reducing the ESMA's time advantage. Overall, the ESMA demonstrated the best comprehensive performance in terms of the minimum and average path lengths, planning time, and stability of the planning results, compared with the SMA, SMA-AGDE, and LRSMA.

Table 1 shows the comparison consequences of the ESMA planning satisfaction rate with that of the other methods. Best results are shown in bold. The satisfaction rate of the other three algorithms did not exceed 70 %, while the ESMA's satisfaction rate in both scenarios was >80%. The satisfaction rate of the ESMA in scenario 1 was higher than that in scenario 2. This shows that the satisfaction rate of the ESMA in scenarios with a punctate obstacle distribution is better than that in scenarios with a banded obstacle distribution. This phenomenon may be due to the higher success rate of the SMA in scenario 1 than in scenario 2. Additionally, the optimization performance of the ESMA in scenario 2 decreased as it is an improvement of the SMA.

**Figure 8:**
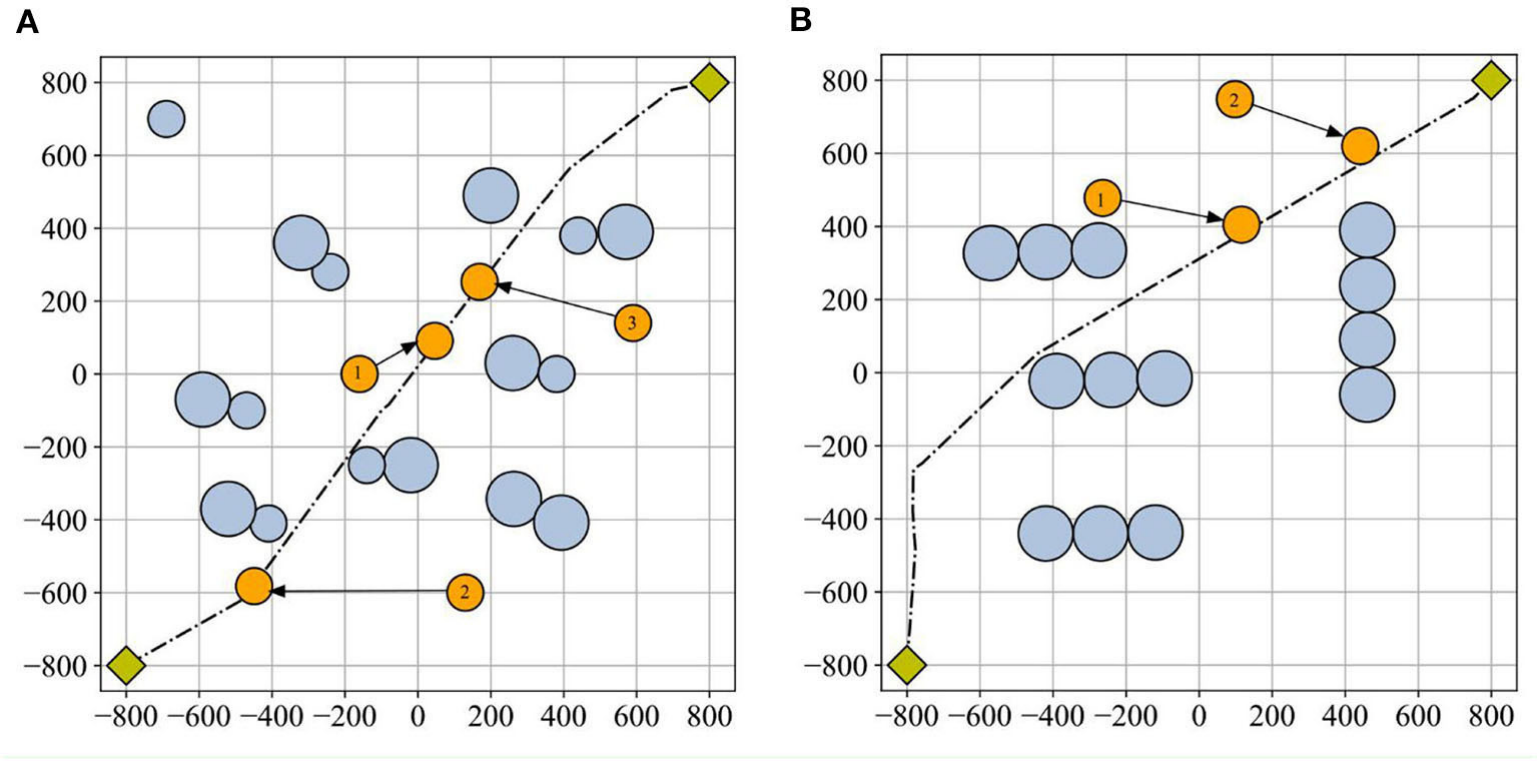
Dynamic obstacles in scenarios: **(A)** scenario 1 and **(B)** scenario 2. The black route represents the path planned by the ESMA in a static environment, orange circles symbolized the dynamic obstacles in the visualization, and each orange circle is numbered to indicate the sequence of the dynamic obstacles. The dynamic obstacles are reset along the original dynamic change path.

The algorithm's sensitivity in a dynamic environment can be assessed by adjusting the obstacle's position and observing the path length before and after the adjustment (Dai et al., 2023). To test the sensitivity of the ESMA, some obstacles in Figure 8 were changed to dynamic obstacles, numbering three in scenario 1 and scenario 2. Therefore, the experiment involved resetting dynamic obstacles along the original dynamic change path. There were two cases: one where the original planned path was not obstructed, and another where it was. As shown in Figure 8, the black route represents the path planned by the ESMA in a static environment, orange circles symbolized the dynamic obstacles in the visualization, and each orange circle is numbered to indicate the sequence of the dynamic obstacles. Table 2 displays all the path length results obtained in all cases. The change rate refers to the relative change in the average length compared to the original path. The average length in scenario 1 is 2,676 cm, while in scenario 2, it is 2,717 cm.

**Table 2:** Comparison results of planned path lengths.

	**Obstacle number**	**Hindered original path (cm)**	**Rate of change**	**Unblocked original path (cm)**	**Rate of change**
Scenario 1	1	2,623	2.00	2,669	0.28
	2	2,753	2.86	2,664	0.47
	3	2,728	1.93	2,661	0.61
Scenario 2	1	2,785	2.51	2,714	0.11
	2	2,788	2.62	2,711	0.21

Table 2 presents the sensitivity analysis of the ESMA algorithm to the position of obstacles. In cases where the obstacle did not obstruct the original planned path, the path length remained unchanged. However, when the obstacle hindered the original planned path, the path length was altered. This analysis highlights the dependence of the path length on the positioning of obstacles and demonstrates the impact of obstacle placement on the effectiveness of the ESMA algorithm.

### 4.3. Robot experiment and analysis

#### 4.3.1. Experimental environment

To further verify the optimization performance of the ESMA, real machine testing was conducted using a robot system. The robot had a length and width of 1.53 and 0.82 m, respectively, and the driving speed was 6–8 km/h, with an Ackerman wire control driving mode. ROS 18.04 was used as the robot's operating system, and the pure pursuit path-tracking method was used to control the robot (Shan et al., 2015). To simplify the calculation, the robot drove at a constant speed of 6 km/h. The test environment used in this study was designed to resemble the simulation environment. It consisted of a blank area where cardboard obstacles were strategically placed.

#### 4.3.2. Robot experiment and analysis

The SMA, SMA-AGDE, LRSMA, and ESMA were run on the robot system to test the 10-run satisfaction. Best results are shown in bold. Table 3 presents various performance metrics for the environment depicted in Figure 5. It includes the minimum path length, average path length, standard deviation of the path length, time required to process the best solution, and average time of path planning. The results indicate that all algorithms were capable of planning collision-free paths in the given environment.

**Table 3:** Performance of the robot using the SMA, SMA-AGDE, LRSMA, and ESMA.

		**Path length (cm)**			**Planning time (s)**	
		**Minimum**	**Mean**	**Standard deviation**	**Processing the best solution**	**Mean**
Scenario 1	SMA	2,455	2,853	336	0.121	**0.115**
	SMA-AGDE	2,489	2,713	**239**	0.156	0.165
	LRSMA	2,395	2,758	302	0.114	0.120
	ESMA	**2,335**	**2,674**	248	**0.104**	**0.115**
Scenario 2	SMA	2,535	2,782	241	0.135	0.131
	SMA-AGDE	2,504	2,916	298	0.176	0.174
	LRSMA	2,473	2,750	227	0.079	0.117
	ESMA	**2,465**	**2,677**	**133**	**0.078**	**0.103**

Best results are shown in bold.

**Figure 9:**
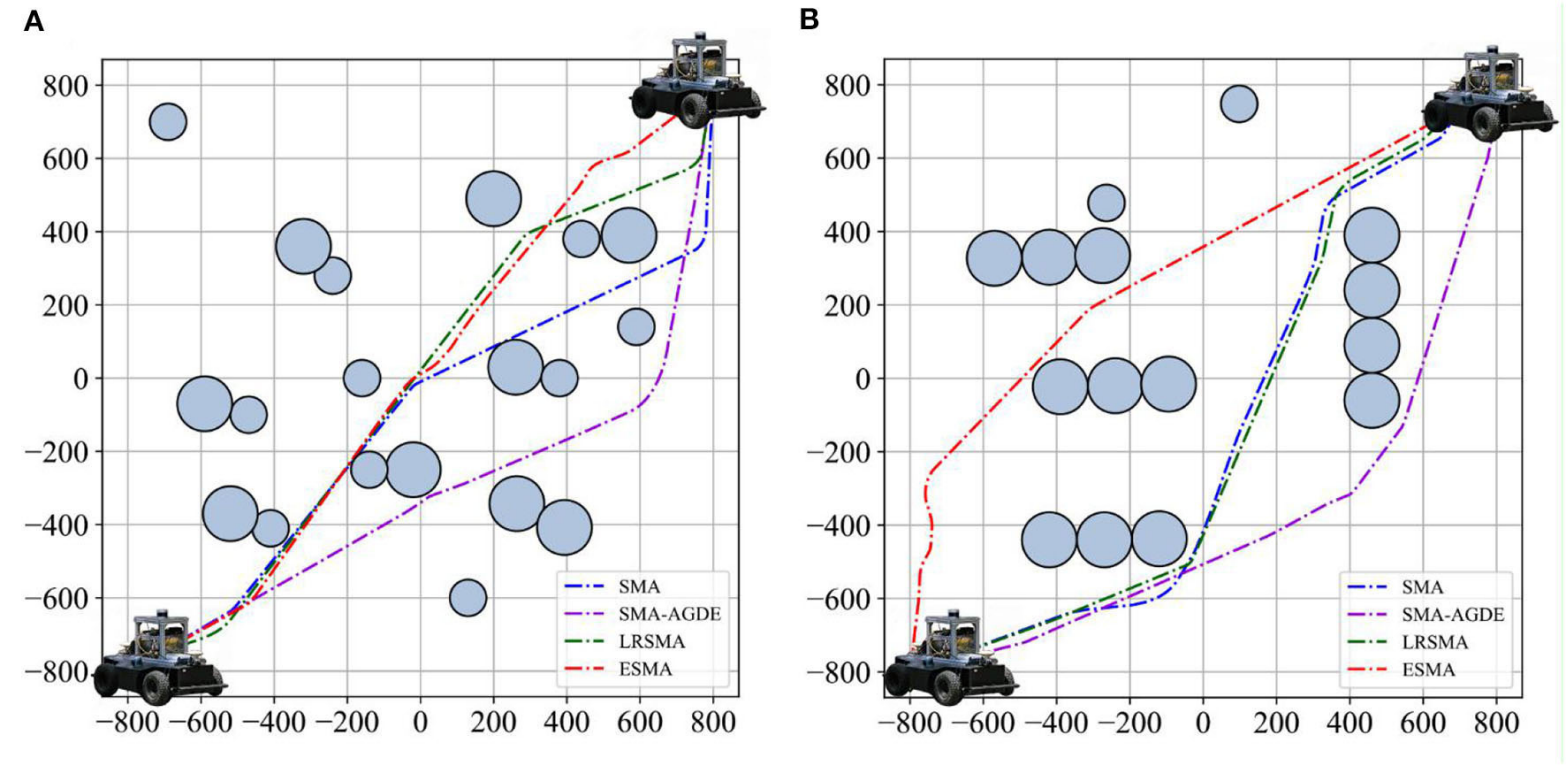
Robot tracking the best solutions in scenarios: **(A)** scenario 1 and **(B)** scenario 2. Four dashed lines stand for the paths of the robot in each picture.

**Figure 10:**
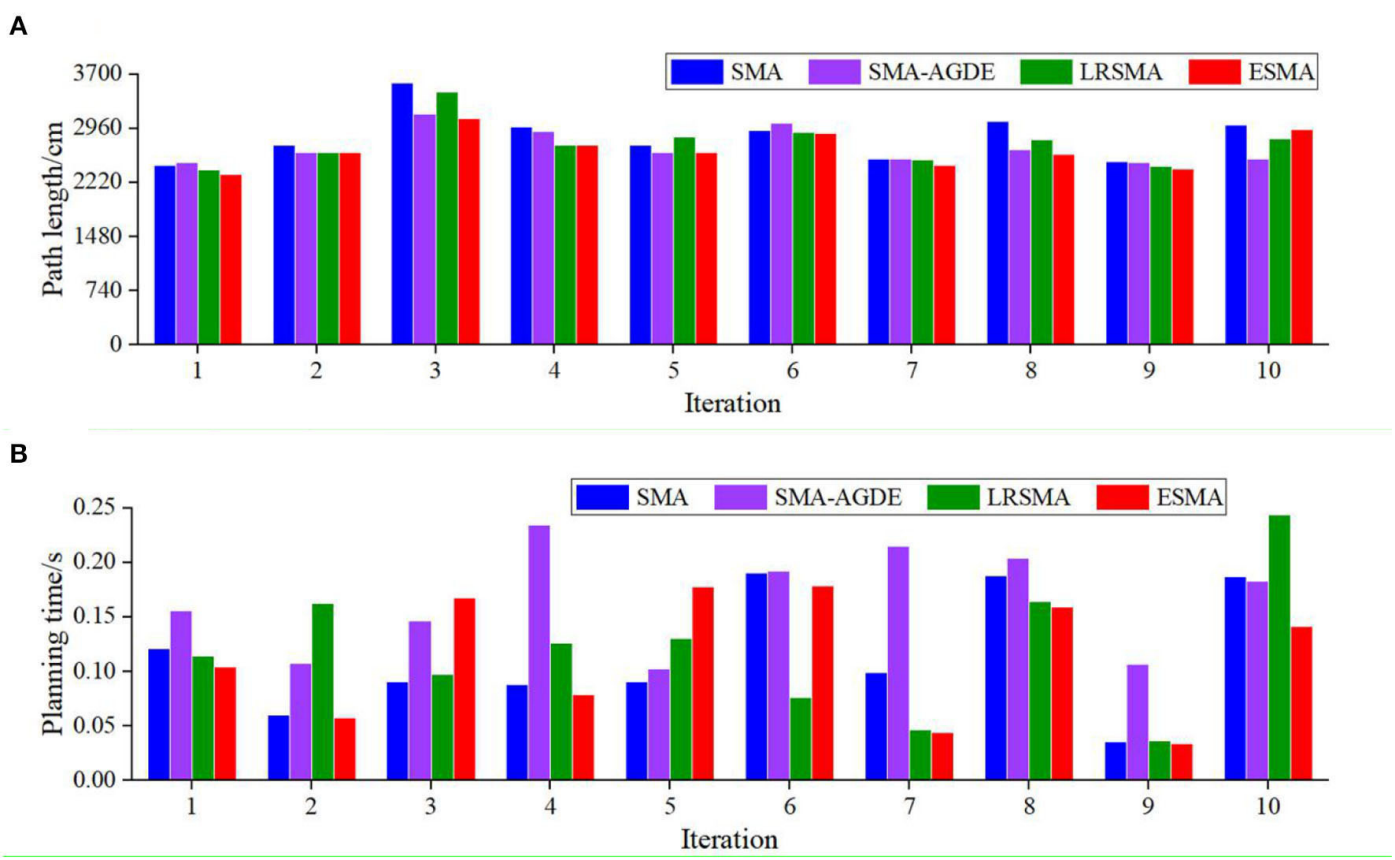
Detail performance of the robot using the four algorithms in scenario 1: **(A)** path length and **(B)** planning time. The height of the bar is the path length in **(A)** and the planning time in **(B)**.

Figure 9A shows the robot tracking the best solutions in scenario 1. Figure 10 shows the detailed path planning lengths and times. From Figure 10A, it can be observed that, in terms of path length, the bars corresponding to the ESMA algorithm have the shortest height, indicating that it finds the shortest path, measuring 2,335 cm. The bar corresponding to the SMA-AGDE algorithm is the tallest, signifying the longest path, measuring 2,489 cm. The bars for ESMA are generally shorter than those for SMA, SMA-AGDE, and LRSMA, implying that only some of ESMA's results are better than those of SMA, SMA-AGDE, and LRSMA. Due to the randomness of the swarm intelligence random algorithm, the planning results show some randomness. Despite the findings mentioned earlier, it is important to note that these specific instances where ESMA did not outperform the other algorithms in terms of path length do not diminish the overall advantages of ESMA in robot path planning. In Figure 10B, it can be seen that the bars corresponding to the ESMA algorithm have the smallest height. As shown in Table 3, compared to the SMA, SMA-AGDE, and LRSMA, the ESMA reduced the minimum path length, average path length, time to process the best solution, and average time by (5.14, 6.60, and 2.57%), (6.69, 1.46, and 3.14%), (16.07, 49.64, and 9.35%), and (0.04, 43.48, and 4.18%), respectively. These results are consistent with the simulation outcomes.

**Figure 11:**
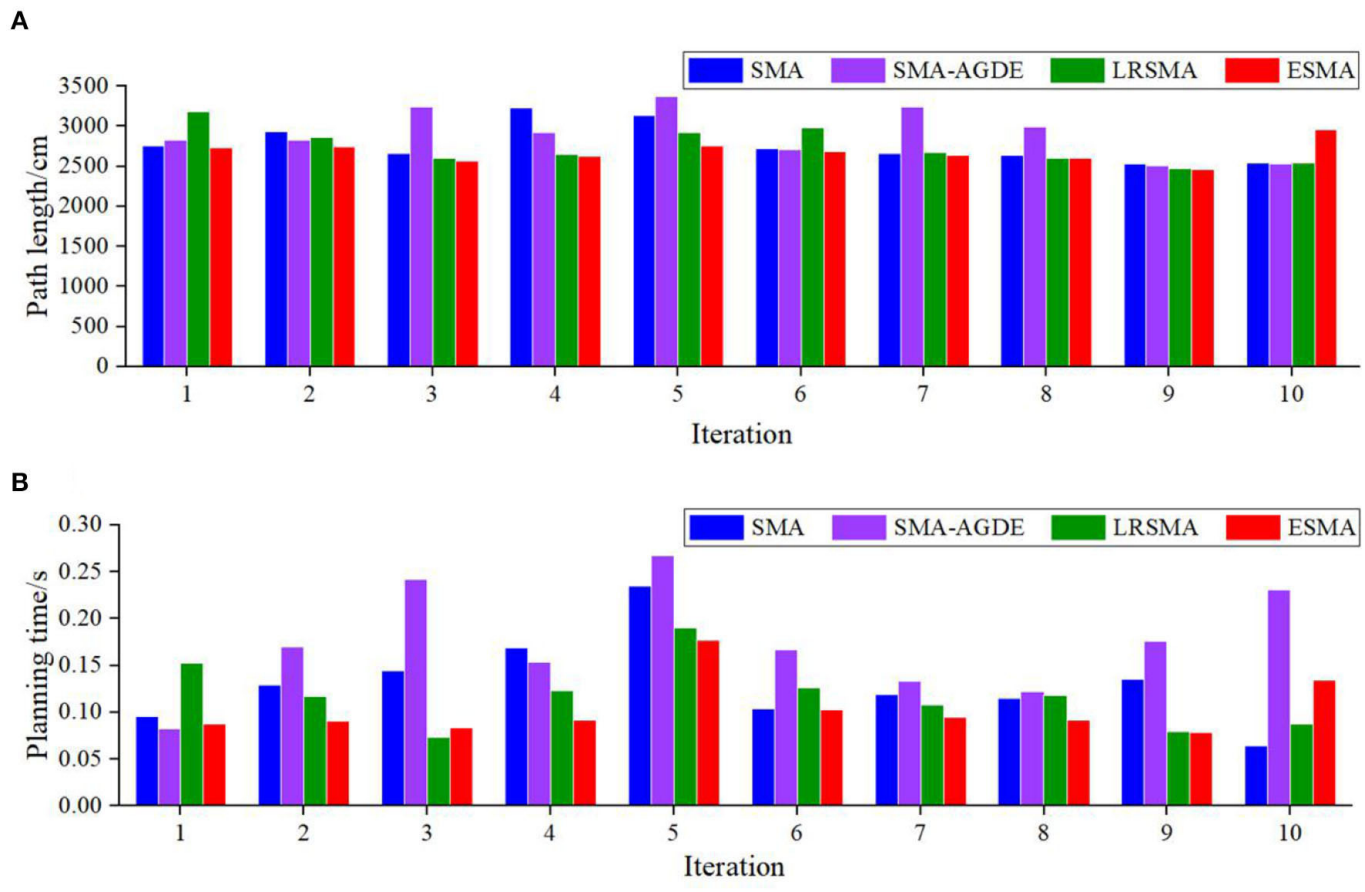
Detailed performance of the robot using the four algorithms in scenario 2: **(A)** path length and **(B)** planning time. The height of the bar is the path length in **(A)** and the planning time in **(B)**.

Figure 9B shows the robot tracking the best solutions in scenario 2. Figure 11 shows the detailed path planning lengths and times. From Figure 11A, it can be observed that, in terms of path length, the bar corresponding to the minimum value achieved by the ESMA algorithm has the smallest height, indicating the shortest path, measuring 2,465 cm. It is relatively close to the minimum value achieved by LRSMA. The bar corresponding to the SMA algorithm is the tallest, signifying the longest path, measuring 2,535 cm. From Figure 11B, it can be seen that ESMA's processing of the best solution is close to LRSMA but outperforms SMA and SMA-AGDE. As depicted in Table 3, compared to the SMA, SMA-AGDE, and LRSMA, the ESMA reduced the minimum path length, average path length, time to process the best solution, and average time by (2.84, 1.58, and 0.32%), (3.92, 8.93, and 2.73%), (73.08, 125.64, and 1.28%), and (27.18, 68.93, and 13.59%), respectively. The results obtained in the physical test environment surpass those achieved in the simulation environment, and this discrepancy could be attributed to the limited number of experiments conducted.

**Figure 12:**
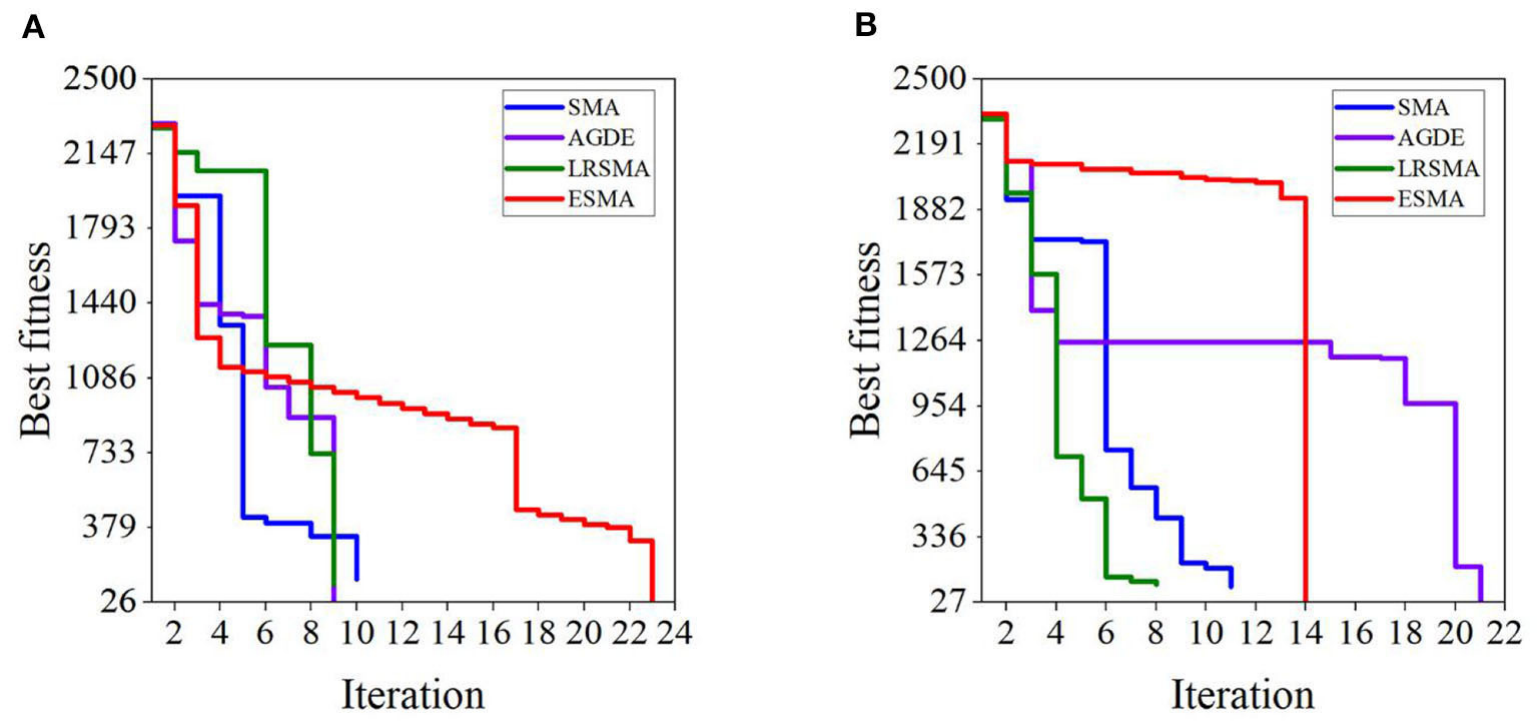
Iteration curves of the four algorithms in scenarios: **(A)** scenario 1 and **(B)** scenario 2. The results are the best fitness values of the four algorithms at the minimum path length. The fitness value is the sum of distances from the current position to the previous position, and from the current position to the endpoint.

Figure 12 compares the convergence speed of the four algorithms at the minimum path length. The fitness value is the sum of distances from the current position to the previous position, and from the current position to the endpoint. The ESMA shows slow convergence because the APF guides the global optimal point to the endpoint with a fixed step size. Fifty iterations in all scenarios are sufficient to achieve good performance.

## 5. Conclusions

This paper introduces the ESMA, a novel swarm intelligence algorithm addressing the path-planning problem in dynamic environments for autonomous mobile robots. The ESMA algorithm incorporates adaptive technology and an artificial potential field to improve the convergence speed while overcoming local optimization issues. In comparison to the SMA, SMA-AGDE, and LRSMA algorithms, ESMA achieved the smallest average minimum path length and minimum path values, along with the shortest algorithm processing time, in experiments conducted in both simulation and real-world scenarios. Consequently, it demonstrated that ESMA is capable of generating shorter collision-free paths with greater accuracy and stability compared to the other solutions. However, on one hand, determining optimal parameters for the Artificial Potential Field (APF) is a challenge, and automated parameter tuning is a topic for future research. On the other hand, enhancing the practical planning stability of ESMA remains an area for further improvement. Future work will address these issues and also involve a comparison of ESMA with other swarm intelligence algorithms in various dynamic environments.

Practically, for ground environments, the ESMA can aid automatic robots or slow automated guided vehicles (AGVs) path planning. For robots, it enables food delivery, express deliveries or automatic sales. For AGVs, it applies to port cargo handling, airport baggage transfer and other relevant applications. However, ESMA may be unsuitable for fast-motion scenarios like relief supplies transportation. Additionally, one of the future research directions is to extend the algorithm's practical application to other environments, such as path planning for unmanned boats, drones, and similar contexts.

## Data availability statement

The original contributions presented in the study are included in the article/supplementary material, further inquiries can be directed to the corresponding author.

## Author contributions

LZ: Conceptualization, Funding acquisition, Validation, Writing—original draft. CH: Writing—review and editing. HS: Validation, Writing—original draft. RC: Validation, Writing—original draft.
